# Plasma proteomic signatures improve risk stratification and personalized screening for gastric cancer

**DOI:** 10.1007/s10120-026-01749-4

**Published:** 2026-05-14

**Authors:** Xue Li, Wen-Hao Shi, Juan Zhu, Ying Chen, Bin Liu, Nai-Ren Zheng, Le Wang, Li Yuan, Ying-Ying Mao, Xiang-Dong Cheng, Ling-Bin Du

**Affiliations:** 1 Department of Cancer Prevention, Zhejiang Cancer Hospital, Hangzhou Institute of Medicine (HIM), Chinese Academy of Sciences, Hangzhou, 310022 Zhejiang China; 2 Zhejiang Key Laboratory of Prevention, Diagnosis and Therapy for Gastrointestinal Cancer, Hangzhou, 310022 Zhejiang China; 3 Analysis Center, Chemistry Department, Tsinghua University, Beijing, 100084 China; 4 Department of Epidemiology, School of Public Health, Nanjing Medical University, Nanjing, 211166 Jiangsu China; 5 Department of Epidemiology, School of Public Health, Zhejiang Chinese Medical University, Hangzhou, 310053 Zhejiang China; 6 Beijing Pineal Diagnostics Company Limited, Beijing, 102206 China; 7 Department of Integrated Chinese and Western Medicine, Zhejiang Cancer Hospital , Hangzhou Institute of Medicine (HIM), Chinese Academy of Sciences, Hangzhou, 310022 Zhejiang China; 8 Department of Gastric Surgery, Zhejiang Cancer Hospital, Hangzhou Institute of Medicine (HIM), Chinese Academy of Sciences, Hangzhou, 310022 Zhejiang China

**Keywords:** Gastric cancer, Proteomics, Biomarker, Cancer screening, Cancer prevention

## Abstract

**Background:**

Accurate identification of individuals at high risk of gastric cancer (GC) remains a major challenge for effective screening. We aimed to identify plasma proteomic signatures and develop a risk prediction model for GC risk stratification.

**Methods:**

Plasma proteomic profiling was performed using liquid chromatography–tandem mass spectrometry in a case–control discovery set (100 GC cases and 94 controls). Candidate proteins were evaluated in 52,552 UK Biobank participants with a median follow-up of 13.63 years, during which 92 incident GC cases were identified. Risk models integrating clinical, genetic, and proteomic factors were developed using LASSO-penalized Cox regression with stability selection and internally validated using bootstrap resampling.

**Results:**

Among 2306 differentially expressed proteins in discovery, 25 were replicated in validation at nominal significance (*P* < 0.05) with consistent directions. Two proteins (CTSD and GGH) remained significant after false discovery rate correction. A primary proteomic model (clinical factors plus five proteins) improved discrimination versus clinical model (optimism-corrected C-index: 0.745 vs. 0.732, *P* = 0.046). Risk stratification revealed a clear GC risk gradient: hazard ratios were 6.08 (95% CI 2.15–17.20) for moderate-risk and 23.88 (95% CI 8.66–65.87) for high-risk groups. The risk score was also associated with GC risk as continuous variable (HR per standard deviation: 1.09, 95% CI 1.08–1.11). The 15-year cumulative incidence ranged from 0.02 to 0.56% across risk groups. Decision curve analysis indicated improved clinical utility.

**Conclusions:**

Plasma proteomic signatures may improve GC risk stratification beyond traditional clinical factors and could support more targeted screening strategies. Further validation is warranted.

**Supplementary Information:**

The online version contains supplementary material available at 10.1007/s10120-026-01749-4.

## Introduction

Gastric cancer presents a significant global health burden, ranking among the top five most prevalent cancers worldwide [1], with particularly high incidence rates in developing countries [2]. Contributing factors to this elevated incidence include *Helicobacter pylori* infection, tobacco use, alcohol consumption, dietary habits, and genetic predisposition [3]. Despite advancements in clinical treatment, gastric cancer is often diagnosed at advanced stages complicating treatment and leading to poor prognosis [4]. Therefore, strengthening prevention and control measures for gastric cancer is essential.

Several strategies have been proposed and implemented for the prevention and control of gastric cancer, including public health education, health management, early screening, and interventions targeting etiological factors [5]. Endoscopic screening and early diagnosis have been shown to significantly reduce both the incidence and mortality of gastric cancer, particularly in high-risk populations [6, 7]. However, the adoption of endoscopic screening for gastric cancer prevention remains challenging in resource-limited areas, and its effectiveness in non-high-risk populations requires further evaluation. Identifying asymptomatic individuals who should be prioritized for gastric cancer screening is crucial.

Circulating proteins, which play key roles in the biological processes leading to carcinogenesis, may reflect pathological conditions and hold promise for early detection, progression monitoring, and prognosis assessment. Techniques such as proteomic profiling have shown potential for identifying novel biomarkers. Several studies suggested that circulating protein biomarkers have the potential to predict cancer risks [8, 9]. Mass spectrometry enables the simultaneous measurement of thousands of proteins, however, many of which have not been previously evaluated in the context of gastric cancer risk. Identifying protein biomarkers associated with gastric cancer onset offers the potential for improved risk stratification, and validation through large-scale longitudinal prospective cohort studies is necessary.

**Fig. 1:**
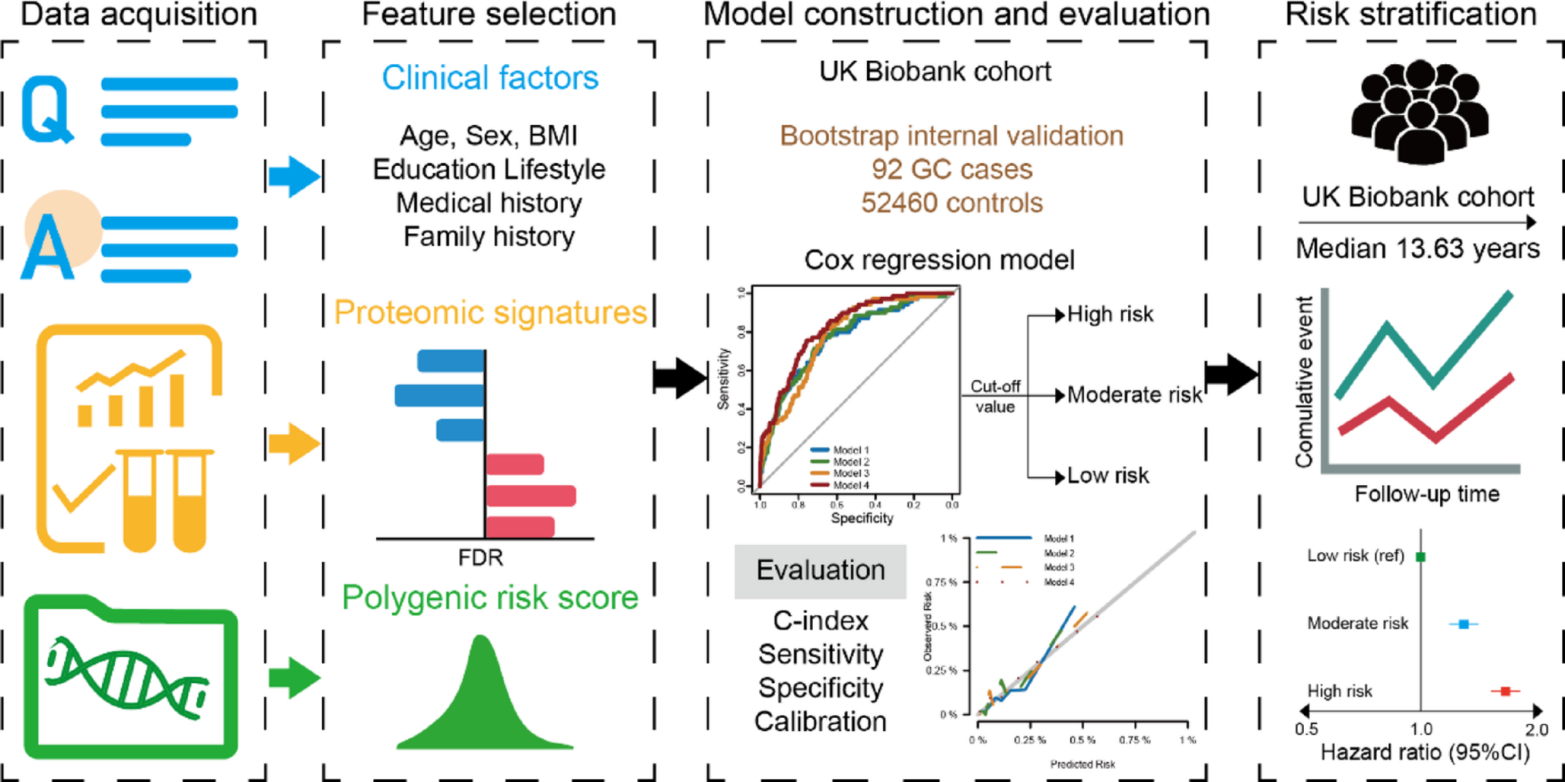
Flowchart of the overall study design. BMI, body mass index; CI, confidence interval

In this study, we employ a two-stage research design, combining a case-control study in the discovery stage and a prospective cohort study in the validation stage. The aim is to identify circulating protein biomarkers associated with gastric cancer and integrate these findings with clinical and genetic factors to enhance gastric cancer risk stratification and enable personalized screening (Fig. 1).

## Methods

### Study population

**Fig. 2:**
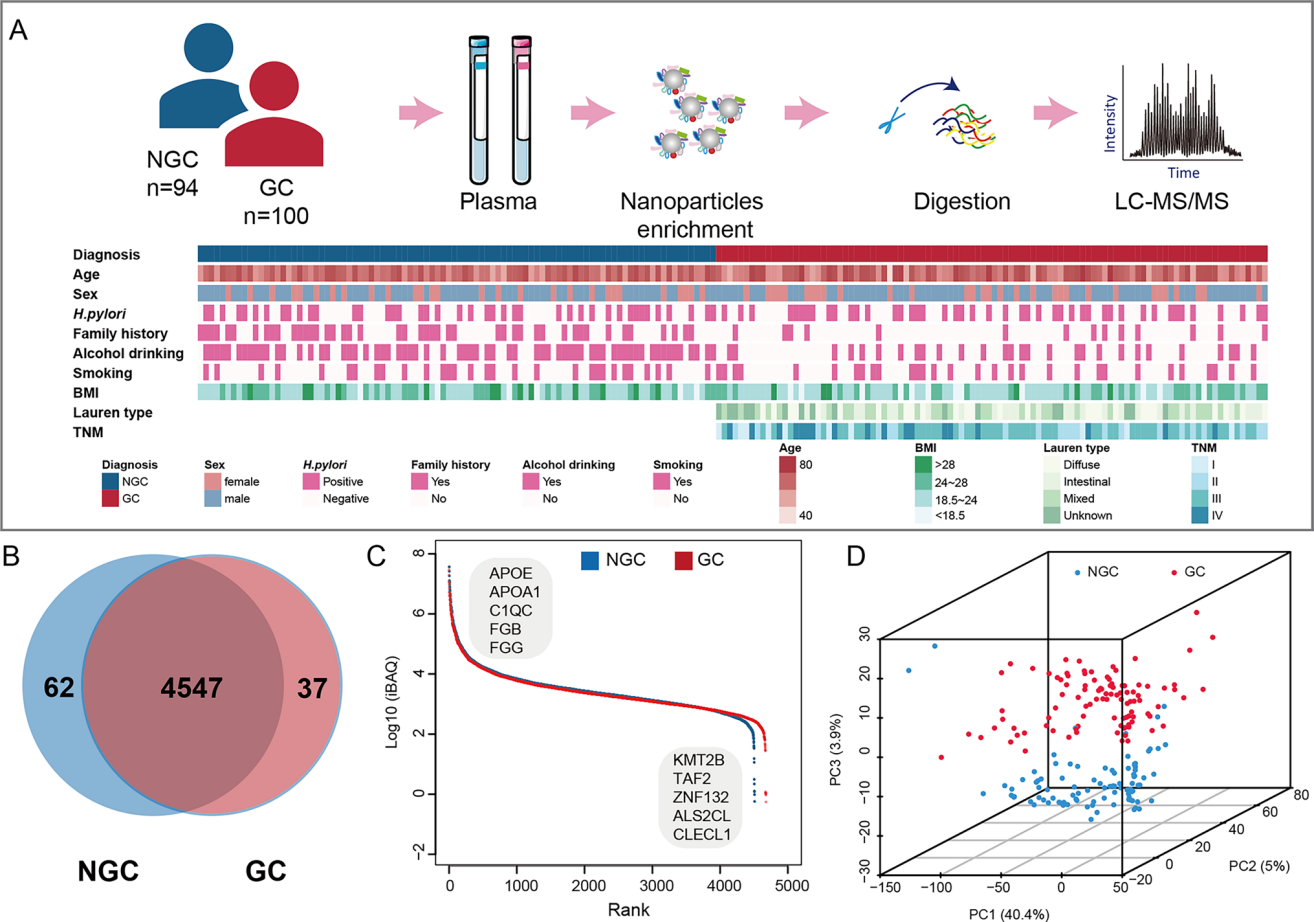
Liquid chromatography-tandem mass spectrometry-based plasma proteomic profiling of gastric cancer and controls in the discovery stage. **A** Overview of plasma proteomics using data-independent acquisition mass spectrometry. **B** Comparison of the number of proteins identified in gastric cancer and non-gastric cancer groups. **C** Proteins identified in gastric cancer and non-gastric cancer groups ranked by median intensity. **D** Principal component analysis of plasma proteomic profiles in gastric cancer and non-gastric cancer groups. GC, gastric cancer; NGC, non-gastric cancer

In the discovery stage, a case-control study was initially designed with 194 participants, comprising individuals with gastric cancer as the case group, and individuals without cancerous lesions as the non-gastric cancer control group. All participants were recruited from Zhejiang Cancer Hospital between 2022 and February 2023. The case group included newly diagnosed gastric cancer patients, who had not undergone prior treatments such as chemotherapy, radiotherapy, targeted therapy, or biological therapy. Patients with two or more malignancies were excluded from the study. Pathological staging followed the 8th edition of the UICC & AJCC TNM classification. The control group comprised healthy individuals who underwent endoscopy as part of routine physical examinations. Participants in this study ranged in age from 32 to 81 years, with 135 males and 59 females. The case group consisted of 100 gastric cancer patients, classified as follows: 10 in stage I, 22 in stage II, 50 in stage III, and 18 in stage IV. No participants with completely normal gastric mucosa were identified. The control group included 16 individuals with superficial gastritis, 31 with chronic atrophic gastritis, and 47 with intestinal metaplasia. Detailed clinical information for the enrolled participants, including age, sex, body mass index, smoking status, alcohol drinking, family history of cancer, Lauren classification, and tumor-node-metastasis staging, is provided in Fig. 2A and Table S1.

The validation cohort was derived from the UK Biobank, with participants recruited between 2006 and 2010. Gastric cancer diagnoses in the UK Biobank cohort were obtained through linkage to hospital data, cancer registries, and death registries, using the International Classification of Diseases (ICD). The time to gastric cancer occurrence was calculated from the cohort entry date until the occurrence of gastric cancer, death, or administrative censoring (October 31, 2022), whichever occurred first. Of the UK Biobank cohort, 92 gastric cancer and 52,460 gastric cancer-free participants with proteomic data were involved in the analysis, with a median (interquartile range) follow-up period of 13.63 (12.90–14.38) years. Baseline characteristics of participants in the validation cohort were shown in Table S2.

### Ethics

The discovery stage study was approved by the Institutional Review Board of Zhejiang Cancer Hospital (No. IRB-2025-368(IIT)). Ethics approval for the UK Biobank study was obtained from the North West Centre for Research Ethics Committee (11/NW/0382). The proteomic profiling of the UK Biobank was approved by the Access Subcommittee of UK Biobank under Access Management System Application No. 65,851. All participants provided written informed consent, and the study was conducted in accordance with the Declaration of Helsinki.

### Sample collection and processing

Fasting blood samples (5 ml) were collected from each participant prior to any gastroscopy or surgery in the discovery stage. The samples were transported to the laboratory within 1 h at 4 °C for processing. Plasma was separated by centrifugation at 1800 g at 4 °C for 10 min, using EDTA as an anticoagulant. After centrifugation, the plasma layer was collected and stored at − 80 °C for further analysis.

### Protein extraction, proteomic profiling, data processing, and protein quantification

Plasma samples were processed using a plasma protein enrichment kit (PH035, Beijing Precise Health Biotechnology Co., Ltd). Briefly, 20 µL of plasma was mixed with 80 µL of enrichment buffer. Subsequently, 20 µL of enrichment magnetic beads were added, and the mixture was incubated at room temperature for 30 min. The enriched sample was washed twice using washing solution. Next, digestion buffer containing 2 µg of trypsin was added, and the solution was vortexed thoroughly. The mixture was incubated at 37 °C overnight for digestion. After digestion, 100 µL of termination buffer was added, and the sample was centrifuged at 16,000 g for 20 min. The supernatant was collected for desalting.

For chromatography, mobile phase A consisted of a 0.1% formic acid (FA) solution, while phase B was 80% acetonitrile (ACN) containing 0.1% FA. Lyophilized peptides were resuspended in 12µL mobile phase A and centrifuged at 16,000 g for 15 min. The supernatant was transferred to a sample vial. 3 µL sample was loaded onto an UHPLC system (UHPLC 3000, Thermo Fisher Scientific, USA) at a flow rate of 3 µL/min. Peptides were separated using a C18 analytical column (1.9 μm, ID150, 30 cm length) at a flow rate of 600 nL/min. Mobile phase B was increased from 8 to 50% in 60 min. The sample was analyzed using a Thermo Scientific Orbitrap Eclipse mass spectrometer (Thermo Fisher Scientific, USA) with a Nanospray Flex ion source. The ion spray voltage was set to 2.2 kV, and the ion transfer tube temperature was set to 320 °C.

The mass spectrometer was operated in Data Independent Acquisition (DIA) mode. The first-stage scan resolution was set to 60,000, with a scan range of 350–1150 m/z and an automatic maximum injection time. The second-stage scan resolution was set to 30,000, with 30 scan windows and a collision energy of 32%. The DIA raw files were processed using Spectronaut 17.4 (Biognosys) software for database searching. The search parameters were set to include trypsin digestion, allowing up to two missed cleavages permitted. Fixed modifications were set to Carbamidomethyl (C), and variable modifications included Oxidation (M) and Acetylation (N-terminal). A false discovery rate (FDR) threshold of less than 1.0% was employed to protein level. The search was conducted against the UniProt human protein database.

The detailed methodology for antibody-based protein profiling using the Olink Explore 3072 platform, including study-wide protein measurements, processing, and quality control procedures, is described in a previous study [10].

### Statistical analyses

#### Identification of plasma proteomic signatures associated with gastric cancer

In the discovery stage, principal component analysis (PCA) was used to visualize the proteomic profiles of subjects with gastric cancer and those without gastric cancer. Differentially expressed proteins were defined as upregulated if the Log_2_ fold change (Log_2_FC) was greater than 0, or downregulated if Log_2_FC was less than 0. Multiple testing correction was performed using the FDR < 0.05 as the significance threshold. In the validation stage, the association between each protein and gastric cancer risk was assessed using Cox proportional hazards regression. Nominal statistical significance (*P* < 0.05) with consistent direction of effect between discovery and validation stages was used as the primary criterion for replication. To further address multiple comparisons, false discovery rate correction was applied across all tested proteins, and proteins with FDR < 0.05 were considered high-confidence findings.

### Polygenic risk score (PRS)

The genotyping, imputation, and quality control procedures employed in the UK Biobank have been described elsewhere [11]. In brief, genotyping was performed by using two similar chips, the UK Biobank Axiom Array and the UK BiLEVE Axiom Array (Affymetrix). Imputation was constructed using the merged UK10K and 1000 Genomes Phase 3. We selected 112 single nucleotide polymorphisms (SNPs) associated with gastric cancer at a genome-wide significant level (*P* < 5 × 10^−8^) from the Finngen R12 (Table S3) [12]. The genotype data for each SNP associated with gastric cancer were obtained from the UK Biobank, and a PRS for gastric cancer was calculated using the following equation: $\:\mathrm{W}\mathrm{e}\mathrm{i}\mathrm{g}\mathrm{h}\mathrm{t}\mathrm{e}\mathrm{d}\:\mathrm{P}\mathrm{R}\mathrm{S}=\sum\:_{i=1}^{n}{}_{i}{SNP}_{i}$. Where SNP_i_ represents the dosage of the effective allele for SNP_i_, β_i_ is the effect estimate for SNP i for gastric cancer derived from previous genome-wide association studies (GWAS), and n is the number of instrumental variables obtained from the GWAS [12] .

### Construction of risk prediction model and risk score

Clinical characteristics of participants in the validation cohort were derived from the UK Biobank dataset. Initially, a univariable Cox proportional hazards regression model was used to explore potential clinical factors associated with gastric cancer risk. A multivariable Cox regression model with backward selection was then applied to identify variables for inclusion in the prediction model. Hazard ratios (HRs) and 95% confidence intervals (CIs) were calculated. Proteins identified as being associated with gastric cancer risk in the two-stage study were further selected using LASSO-penalized Cox regression. To reduce the risk of overfitting given the limited number of gastric cancer events, stability variable selection among validated proteins was performed using LASSO-penalized Cox regression with 200 bootstrap iterations; each iteration randomly drew 80% of the sample without replacement. In each subsample, LASSO-Cox regression with 10-fold cross-validation was conducted, and variables selected at lambda.1se were recorded. The selection frequency for each variable was calculated across all iterations. Proteins with a selection frequency ≥ 0.7 were considered stable predictors and were included in the primary protein model. Proteins with a selection frequency ≥ 0.4 were selected to construct an exploratory extended protein model. Four prediction models were constructed in the validation cohort using the Cox proportional hazards model, including a clinical model (Model 1), a clinical model plus polygenic risk score (Model 2), a primary protein model incorporating clinical factors and stable proteomic biomarkers (selection frequency ≥ 0.7) (Model 3), and an exploratory extended protein model incorporating additional proteomic biomarkers (selection frequency ≥ 0.4) (Model 4).

Internal validation was performed using 1000 bootstrap resamples to estimate optimism-corrected performance, including the C-index and calibration slopes. Shrinkage factors were calculated to quantify the degree of potential overfitting. Model discrimination was assessed using optimism-corrected C-index and time-dependent ROC curves. C-index was compared using the ‘compareC’ function in R. Calibration plots were generated to assess the agreement between predicted and observed gastric cancer-free probabilities. A weighted risk score was calculated for all participants in the validation cohort based on the combination of clinical and proteomic signatures using the ‘predict’ function in R. The formula for the risk score is as follows: Risk Score = exp (β_1_⋅X_1_ + β_2_⋅X_2_+…+β_n_⋅X_n_) where X_n_ represents the level of each variable, and β_n_​ is the corresponding coefficient derived from the Cox regression model. Cut-off values were determined using Youden index. In addition to hazard ratios, absolute cumulative incidence estimates at 5, 10, and 15 years were calculated for each risk group using the Kaplan-Meier method. The distribution of gastric cancer events across risk groups was also reported to complement the relative risk estimates.

Decision curve analysis was conducted to compare the net benefits of the different models.

All analyses were performed using R (version 4.5.3), unless otherwise specified.

## Results

### Constructing a gastric cancer plasma proteome atlas based on mass spectrometry

The flowchart of proteomic profiling and the characteristics of the subjects in the discovery stage are shown in Fig. 2A and Table S1. A total of 4646 highly reliable plasma proteins were identified, of which, 4547 proteins were detected in both gastric cancer and non-gastric cancer groups (Fig. 2B). The protein abundance spanned across 10 orders of magnitude (Fig. 2C). Distinct plasma proteomic profiles were observed between subjects with gastric cancer and those in the non-gastric cancer group, as demonstrated by the PCA analysis (Fig. 2D).

### Plasma proteomic signatures associated with gastric cancer

**Fig. 3:**
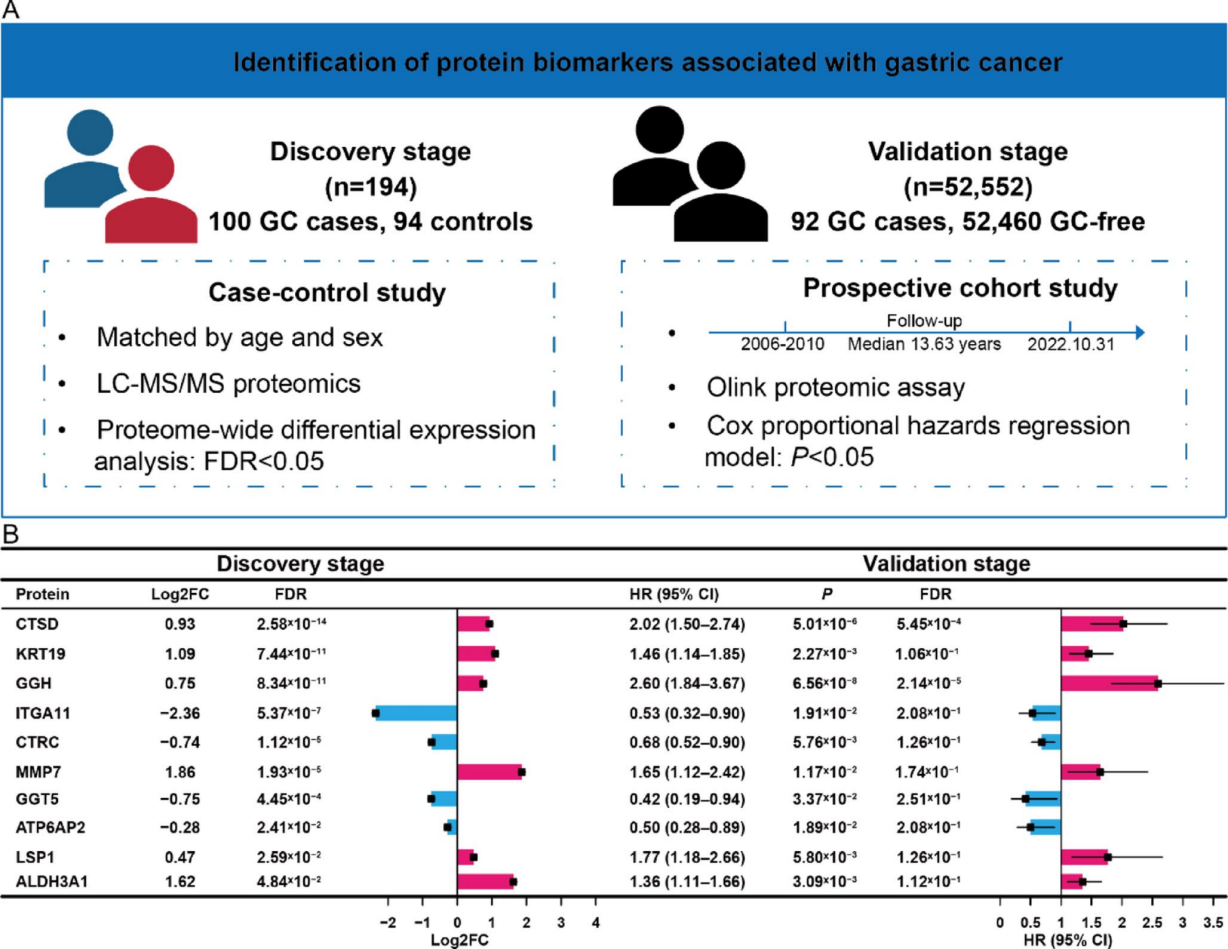
Identification of protein biomarkers associated with gastric cancer. **A** Flowchart of the two-stage study design. **B** Proteins included in the risk prediction models. CI, confidence interval; FC, fold change; FDR, false discovery rate; GC, gastric cancer; HR, hazard ratio; LC-MS/MS, liquid chromatography tandem mass spectrometry

Proteomic signatures were identified and validated through a two-stage study design (Fig. 3A). In the discovery stage, a total of 2306 significant differentially expressed plasma proteins were identified between gastric cancer cases and non-gastric cancer controls, including 881 up-regulated proteins (Log_2_FC > 0, FDR < 0.05) and 1425 down-regulated proteins (Log_2_FC < 0, FDR < 0.05). Among these, 326 proteins were available in the UK Biobank validation cohort and were further evaluated. Twenty-five proteins were replicated at nominal significance (*P* < 0.05) with consistent directions. Among them, 20 proteins were positively associated with the risk of gastric cancer (HR > 1, *P* < 0.05), while 5 proteins showed an inverse association (HR < 1, *P* < 0.05) (Table S4). After controlling for multiple testing using the false discovery rate, two proteins (CTSD and GGH) remained statistically significant (FDR < 0.05) and were recognized as high-confidence proteins, indicating robust associations (Fig. 3B). Proteins showing nominal replication (*P* < 0.05) were carried forward for model development. LASSO-penalized Cox regression was applied to further select predictive features. As a result, five proteins (CTSD, KRT19, CTRC, ALDH3A1, and GGH) were selected to construct the primary prediction model (selection frequency ≥ 0.7), while an additional five proteins (MMP7, LSP1, GGT5, ITGA11, and ATP6AP2) were included to build an exploratory extended model (selection frequency ≥ 0.4) (Fig. 3B).

### Construction of prediction models

**Fig. 4:**
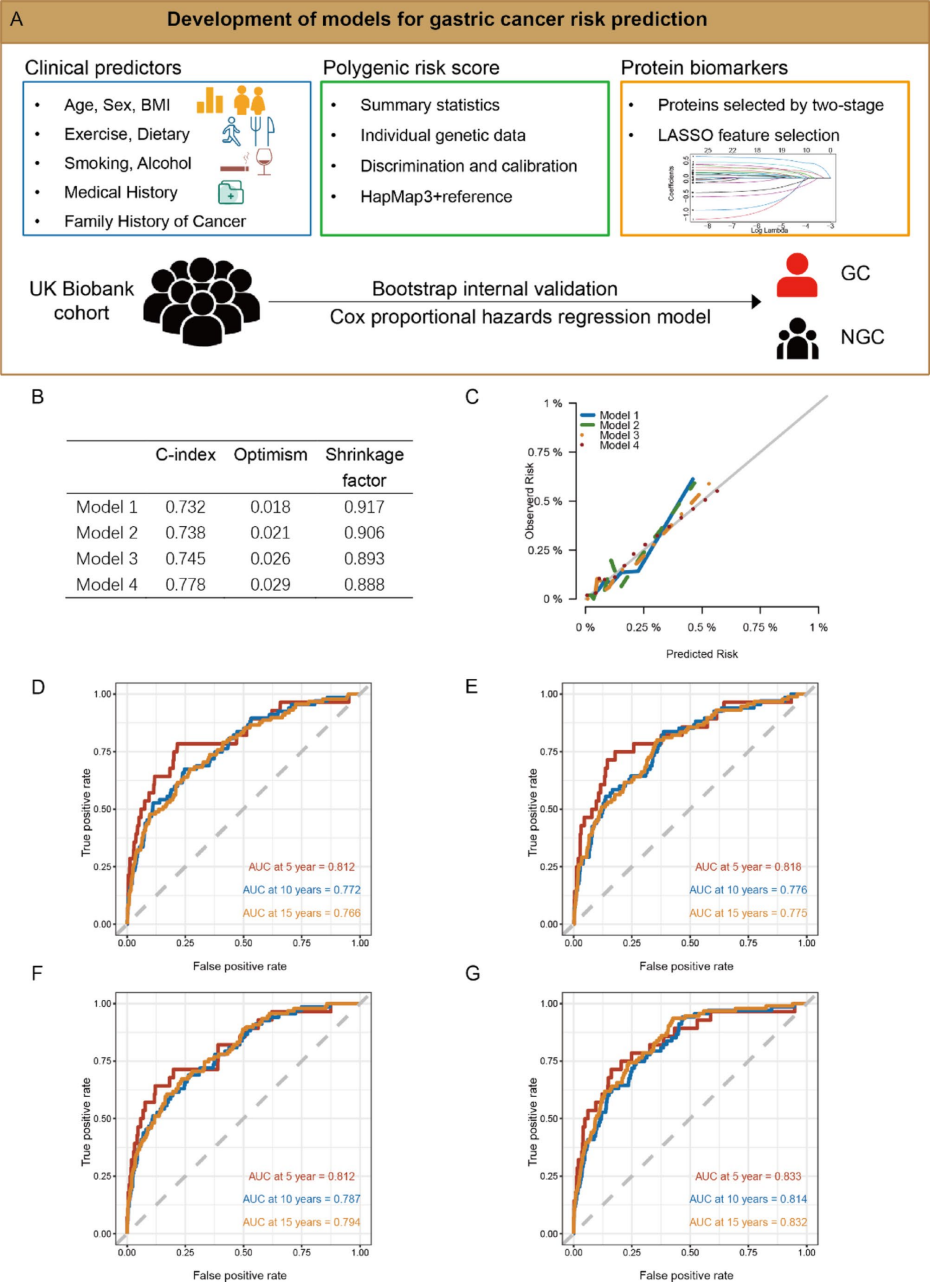
Development of models for gastric cancer risk prediction. **A** Schematic overview of the prediction model, including parameters incorporated and the workflow for model construction and internal validation. **B** Optimism-corrected C-index, optimism, and shrinkage factors for the four prediction models. **C** Calibration curves showing the agreement between predicted and observed probabilities of gastric cancer risk for the four models. **D**–**G** Time-dependent receiver operating characteristic curves for the clinical model (**D**), clinical plus polygenic risk score model (**E**), clinical plus five-protein model (**F**), and clinical plus ten-protein model (**G**) in the validation cohort. AUC, area under curve; GC, gastric cancer; PRS, polygenic risk score.

A total of 52,552 participants were included in the UK Biobank validation cohort and used for model construction, with a median follow-up of 13.63 years (interquartile range: 12.90–14.38). During this period, 92 cases of gastric cancer were identified (Fig. 4A). Four Cox proportional hazards models were developed to evaluate the incremental predictive value of genetic and proteomic markers. The baseline clinical model (Model 1), including age, sex, education, smoking status, physical activity, and family history of cancer, demonstrated an optimism-corrected C-index of 0.732. The addition of the polygenic risk score (Model 2) resulted in a modest improvement in discrimination (C-index: 0.738, *P* = 0.084). Incorporation of proteomic markers further improved model performance. The primary protein model (Model 3), which included clinical factors and five proteins (CTSD, KRT19, CTRC, ALDH3A1 and GGH) selected through stability selection (frequency ≥ 0.7), achieved an optimism-corrected C-index of 0.745 (*P* = 0.046). The extended protein model (Model 4), including ten proteins (CTSD, KRT19, CTRC, ALDH3A1, GGH, MMP7, LSP1, GGT5, ITGA11 and ATP6AP2), showed the highest discrimination with an optimism-corrected C-index of 0.778 (*P* < 0.001). Given the limited number of events, this extended model is presented as an exploratory analysis and may be more prone to overfitting. Bootstrap-estimated optimism ranged from 0.018 to 0.029 across models, and shrinkage factors ranged from 0.89 to 0.92, indicating acceptable levels of overfitting (Fig. 4B). Calibration plots showed good agreement between predicted and observed risks for all models (Fig. 4C). Time-dependent ROC analyses at 5, 10, and 15 years consistently demonstrated improved discrimination with the inclusion of proteomic markers (Fig. 4D–G).

### Risk stratification for gastric cancer screening

**Fig. 5:**
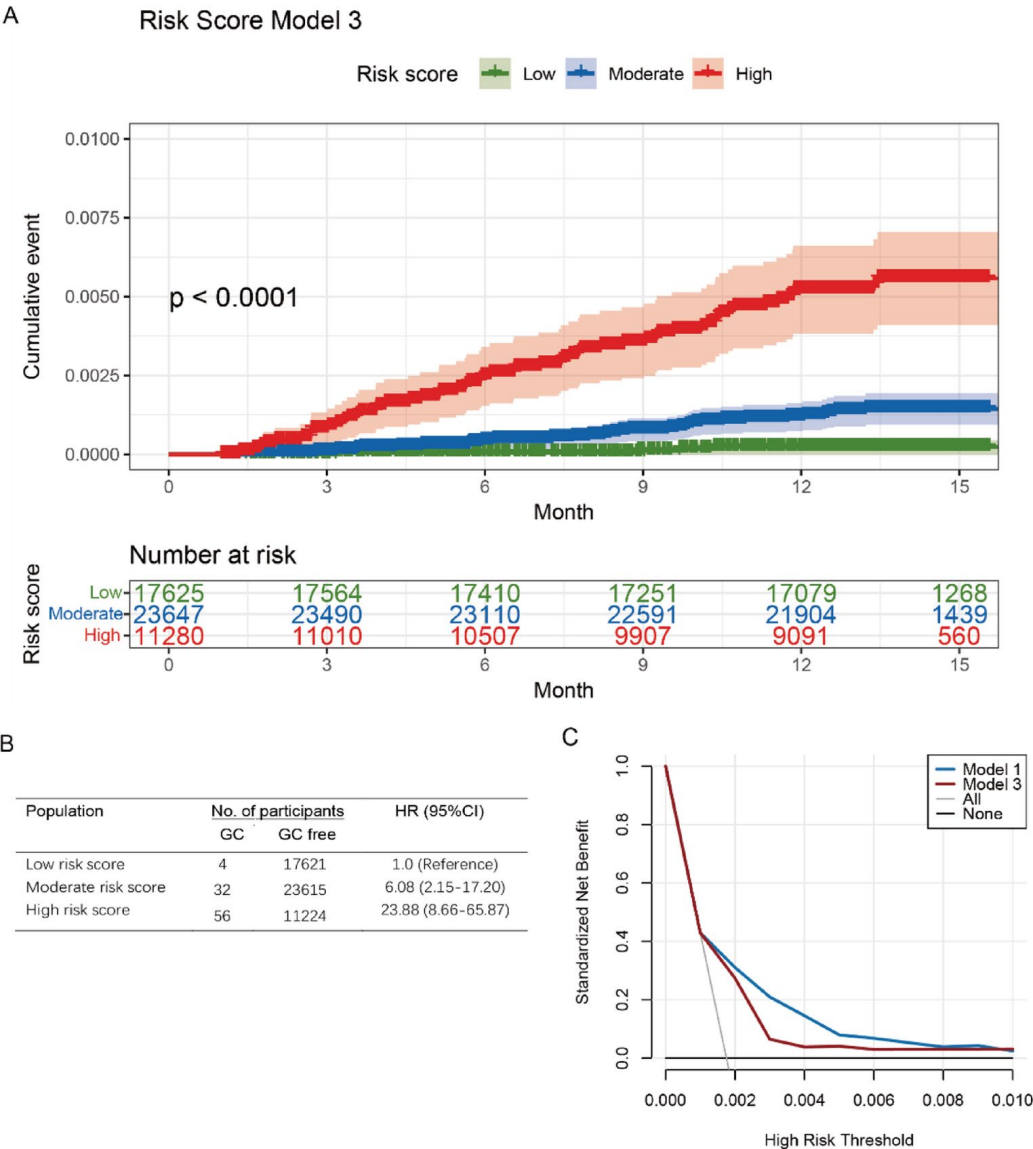
Risk stratification of gastric cancer screening in the validation cohort. **A** Cumulative incidence of gastric cancer according to risk score categories. **B** Hazard ratios for gastric cancer across different risk groups compared with the low-risk group. **C** Decision curve analysis showing net benefit of different risk prediction models. CI, confidence interval; HR, hazard ratio

Risk stratification was performed using the primary protein model (Model 3). Participants were categorized into low-, moderate-, and high-risk groups based on the distribution of the risk score. A clear gradient in gastric cancer risk was observed across the three groups (log-rank *P* < 0.0001, Fig. 5A). Compared with the low-risk group, the HR was 6.08 (95% CI 2.15–17.20) for the moderate-risk group and 23.88 (95% CI 8.66–65.87) for the high-risk group (Fig. 5B). This strong relative effect corresponded to a marked concentration of gastric cancer cases in the high-risk group. Specifically, 56 of 92 events (60.9%) occurred in the high-risk group, whereas only 4 events (4.4%) occurred in the low-risk group (Fig. 5B). Absolute risk estimates further demonstrated a clear separation across groups. The estimated 15 year cumulative incidence of gastric cancer was approximately 0.02%, 0.14%, and 0.56% in the low-, moderate-, and high-risk groups, respectively. When analyzed as a continuous variable, the risk score was significantly associated with gastric cancer risk, with a hazard ratio (95%CI) of 1.09 (1.08–1.11) per standard deviation increase (*P* < 0.001), indicating a more moderate and stable effect size. Decision curve analysis demonstrated that the model incorporating proteomic markers (Model 3) provided a higher net benefit across a wide range of threshold probabilities compared with the clinical model (Model 1) (Fig. 5C).

## Discussion

In this study, we identified and validated plasma proteomic signatures associated with incident gastric cancer and developed a multi-modal risk prediction model integrating clinical, genetic, and proteomic factors. Using a two-stage design combining mass spectrometry–based discovery and large-scale prospective validation in the UK Biobank cohort, we identified 25 proteins associated with gastric cancer risk, of which two (CTSD and GGH) remained significant after multiple testing correction. The addition of proteomic markers to clinical factors resulted in a modest improvement in discrimination and enabled meaningful risk stratification, with a clear gradient in both relative and absolute risk across groups. Despite the limited number of events, internal validation suggested acceptable model performance and potential clinical utility.

Previous study based on the UK Biobank Pharma Proteomics Project demonstrated that protein signatures can improve the prediction of disease onset across common and rare diseases [13]. The two-stage study design identified and validated 25 circulating protein biomarkers associated with gastric cancer incidence. Several of these proteins have previously been implicated in gastric cancer or other malignancies through diverse molecular mechanisms, including alterations in protein abundance (e.g., MMP7, FABP5) [14–16], mRNA expression levels (e.g., CTSD, GGH) [17, 18], and dysregulation of immune and inflammatory pathways (e.g., LGALS1, LSP1) [19, 20]. MMP7 (matrix metalloproteinase-7) has been extensively reported to facilitate tumor invasion and metastasis in gastrointestinal cancers, underscoring its potential as a diagnostic and prognostic biomarker in gastric cancer [14]. FABPs (fatty acid-binding proteins), which transport long-chain fatty acids (LCFAs), play crucial roles in cancer cell proliferation, metabolism, migration, and progression [16]. Specially, FABP5 is significantly overexpressed in gastric cancer cells and tissues compared to normal controls, potentially modulating immune-related signaling pathways and contributing to tumorigenesis [15]. CTSD (cathepsin D), a lysosomal aspartic protease, undergoes post-translational modification through glycosylation. Aberrant glycosylation of its precursor form impair its normal enzymatic function and is associated with the pathogenesis of several cancers, including gastric cancer [17]. GGH (gamma-glutamyl hydrolase) regulates intracellular folate concentrations needed for cell proliferation, DNA synthesis, methylation, and repair [18, 21]. High GGH expression predicts poor outcomes in stage II/III gastric cancer patients undergoing postoperative adjuvant chemotherapy with S-1^18^. Given the still incomplete understanding of the mechanisms underlying gastric cancer, these differential proteins may represent key molecular events in the disease’s development, offering valuable insights into its pathogenesis.

An individualized, risk-based approach has gained increasing recognition as a promising strategy for the early detection of gastric cancer, highlighting the importance of integrating diverse risk factors, including clinical variables, inherited genetic variants, and omics-based biomarkers—particularly circulating protein markers [22]. Several prediction models based solely on clinical factors have demonstrated C-statistics higher than 0.7 in validation cohorts, supporting the utility of clinical predictors for risk stratification [23–26]. Polygenic risk scores have also been explored, with reported C-statistics ranging from 0.56 to 0.78, although their incremental predictive value appears modest when used alone and may benefit from integration with non-genetic factors [27, 28]. Beyond genetic and clinical predictors, recent research has explored the use of molecular biomarkers for early detection. For example, Li et al. developed a protein-based (CDHR2, ICAM4, PTPRM, CDC27, and FLT1) model for the early diagnosis of cardia gastric cancer, demonstrating promising diagnostic performance [29]. However, most existing studies have focused on diagnostic settings rather than prospective risk prediction in population-based cohorts. In the present study, using data from the UK Biobank cohort, we developed and internally validated multiple risk prediction models. The clinical model demonstrated moderate discrimination (optimism-corrected C-index: 0.732), with only a modest improvement after incorporating polygenic risk scores (C-index: 0.738). In contrast, the addition of circulating proteomic markers resulted in a consistent improvement in discrimination, with the primary protein model achieving an optimism-corrected C-index of 0.745. Although the magnitude of improvement was moderate, the proteomic model enabled effective risk stratification, with clear gradients in both relative and absolute gastric cancer risk across groups, as well as improved clinical utility in decision curve analysis. Collectively, these findings suggest that proteomic data may provide complementary information beyond traditional clinical and genetic factors for gastric cancer risk prediction.

Risk stratification is critical for personalized cancer prevention and population-level screening strategies. A study based on China’s National Screening Program developed a pragmatic model for gastric cancer risk prediction, showing markedly increasing incidence across risk groups [30]. Similarly, pre-screening risk assessment strategies have been proposed to improve resource efficiency and identify high-risk individuals for targeted screening [31]. In the present study, we applied the clinical factors combined with proteomic markers model to the full UK Biobank cohort to generate individual risk scores. Participants were categorized into low-, moderate-, and high-risk groups, and a clear gradient in gastric cancer risk was observed across categories (log-rank *P* < 0.0001). Compared with the low-risk group, the hazard ratios were 6.08 for the moderate-risk group and 23.88 for the high-risk group. Notably, 60.9% of incident cases occurred in the high-risk group, whereas only 4.4% were observed in the low-risk group, indicating substantial risk concentration. Absolute risk estimates further supported this pattern, with 15 year cumulative incidence ranging from 0.02 to 0.56% across risk groups. Although the absolute risks were low, reflecting the low baseline incidence of gastric cancer in the UK Biobank population, a clear and clinically meaningful gradient was observed, indicating effective risk stratification. These findings suggest that the proposed model may enable meaningful risk stratification at the population level. In addition, decision curve analysis indicated that the proteomic model may provide greater net clinical benefit than models based on clinical factors alone. Together, these results support the potential value of circulating protein biomarkers in improving risk stratification and informing more targeted screening strategies.

This study has several strengths. The two-stage design, combining unbiased proteomic discovery with large-scale prospective validation in the UK Biobank cohort, enhances the robustness of the findings. The application of LASSO penalization with stability selection enabled parsimonious model construction and reduced the risk of overfitting under limited events-per-variable conditions. In addition, internal validation using bootstrap resampling provided optimism-corrected performance estimates, further supporting the reliability of the model. The integration of proteomic markers with clinical factors resulted in a modest but consistent improvement in discrimination and enabled effective risk stratification in a population-based setting.

Several limitations should be considered. First, the discovery stage was based on a hospital-based case–control design with a relatively small sample size, and residual confounding cannot be excluded. Second, only proteins measured by the Olink platform were available in the validation cohort, which may have limited the inclusion of additional relevant biomarkers. Third, despite the large sample size, the number of gastric cancer events was relatively small, which may affect the precision of effect estimates, although this was partially addressed through penalized regression and bootstrap validation. Fourth, the model was internally validated but lacks external validation in independent populations, which may limit its generalizability. Finally, the biological mechanisms underlying the identified protein associations were not fully explored.

In conclusion, plasma proteomic signatures may improve risk stratification for gastric cancer beyond traditional clinical factors. Although the improvement in predictive performance was modest, the model was able to identify individuals at substantially elevated risk and may support more targeted screening strategies. Further external validation and mechanistic studies are warranted.

## Electronic Supplementary Material

Below is the link to the electronic supplementary material.

Supplementary Material 1.

## Data Availability

The data that support the findings of this study are available from the corresponding author upon reasonable request.
