# Supplementary material for: Plasma proteomic signatures improve risk stratification and personalized screening for gastric cancer
Source: Gastric Cancer. 2026 May 14;29(4):725–36. doi: 10.1007/s10120-026-01749-4 (PMC13315260; doi:10.1007/s10120-026-01749-4)
Supplement: Supplementary file 1 — Supplementary Material 1. [file 10120_2026_1749_MOESM1_ESM.docx]

Table S1 Characteristics of participants in the discovery stage

| Characteristic | All (n=194) | Non-gastric cancer (n=94) | Gastric cancer (n=100) |
| --- | --- | --- | --- |
| Age, median (interquartile range) | 65.0 (58.0-69.0) | 64.0 (59.0-67.8) | 65.0 (58.0-70.0) |
| Sex (n, %) |  |  |  |
| Male | 135 (69.6) | 66 (70.2) | 69 (69.0) |
| Female | 59 (30.4) | 28 (29.8) | 31 (31.0) |
| Body mass index (n, %) |  |  |  |
| <18.5 kg/m^2^ | 18 (9.3) | 6 (6.4) | 12 (12.0) |
| 18.5~24 kg/m^2^ | 100 (51.5) | 46 (48.9) | 54 (54.0) |
| 24~28 kg/m^2^ | 57 (29.4) | 29 (30.9) | 28 (28.0) |
| ≥28 kg/m^2^ | 19 (9.8) | 13 (13.8) | 6 (6.0) |
| Smoking (n, %) |  |  |  |
| No | 134 (69.1) | 64 (68.1) | 70 (70.0) |
| Yes | 60 (30.9) | 30 (31.9) | 30 (30.0) |
| Alcohol Drinking (n, %) |  |  |  |
| No | 101 (52.1) | 29 (30.9) | 72 (72.0) |
| Yes | 93 (47.9) | 65 (69.1) | 28 (28.0) |
| Family History (n, %) |  |  |  |
| No | 135 (69.6) | 49 (52.1) | 86 (86.0) |
| Yes | 59 (30.4) | 45 (47.9) | 14 (14.0) |
| *Helicobacter pylori* (n, %) |  |  |  |
| Negative | 107 (55.2) | 52 (55.3) | 55 (55.0) |
| Positive | 87 (44.8) | 42 (44.7) | 45 (45.0) |
| TNM staging |  |  |  |
| I | N/A | N/A | 10 (10.0) |
| II | N/A | N/A | 22 (22.0) |
| III | N/A | N/A | 50 (50.0) |
| IV | N/A | N/A | 18 (18.0) |
| Lauren type |  |  |  |
| Diffuse | N/A | N/A | 26 (26.0) |
| Intestinal | N/A | N/A | 26 (26.0) |
| Mixed | N/A | N/A | 32 (32.0) |
| Unknown | N/A | N/A | 16 (16.0) |

Table S2 Baseline characteristics of participants in the validation cohort

| Characteristic | All participants | Gastric cancer-free | Gastric cancer |
| --- | --- | --- | --- |
| Number of participants | 52,552 | 52,460 | 92 |
| Follow-up (years), median (interquartile range) | 13.6 (12.9-14.4) | 13.6 (12.9-14.4) | 7.5 (4.0-10.1) |
| Sex (n, %) |  |  |  |
| Female | 28,379 (54.0) | 28,353 (54.0) | 26 (28.0) |
| Male | 24,173 (46.0) | 24,107 (46.0) | 66 (72.0) |
| Age (years), median (interquartile range) | 58 (50.0-64.0) | 58 (50.0-64.0) | 63 (58.0-66.0) |
| Ethnicity (n, %) |  |  |  |
| White | 49,012 (93.3) | 48,928 (93.3) | 84 (91.3) |
| Non-white | 3,283 (6.2) | 3,275 (6.2) | 8 (8.7) |
| Missing | 257 (0.5) | 257 (0.5) | 0 (0) |
| Education (n, %) |  |  |  |
| College or university degree | 16,781 (32.0) | 16,760 (32.0) | 21 (23.0) |
| Below college | 25,643 (49.0) | 25,607 (49.0) | 36 (39.0) |
| Missing | 10,128 (19.0) | 10,093 (19.0) | 35 (38.0) |
| Townsend deprivation index, median (interquartile range) | -2.1 (-3.6-0.8) | -2.1 (-3.6-0.8) | -1.2 (-3.0-1.9) |
| Household income (n, %) |  |  |  |
| Less than 18,000 | 10,741 (20.4) | 10,714 (20.4) | 27 (29.3) |
| 18,000 to 30,999 | 11,549 (22.0) | 11,529 (22.0) | 20 (21.7) |
| 31,000 to 51,999 | 11,219 (21.3) | 11,203 (21.4) | 16 (17.4) |
| 52,000 to 100,000 | 8,562 (16.3) | 8,556 (16.3) | 6 (6.5) |
| More than 100,000 | 2,297 (4.4) | 2,295 (4.4) | 2 (2.2) |
| Missing | 8,184 (15.6) | 8,163 (15.6) | 21 (22.8) |
| Body mass index (n, %) |  |  |  |
| ＜18.5 kg/m^2^ | 268 (0.5) | 267 (0.5) | 1 (1.1) |
| 18.5 ~ 25 kg/m^2^ | 16,722 (32.0) | 16,703 (31.8) | 19 (20.7) |
| 25 ~ 30 kg/m^2^ | 22,458 (43.0) | 22,413 (42.7) | 45 (48.9) |
| ≥30 kg/m^2^ | 12,849 (24.0) | 12,822 (24.4) | 27 (29.3) |
| Missing | 255 (0.5) | 255 (0.5) | 0 (0) |
| Smoking status (n, %) |  |  |  |
| Never smoker | 28,452 (54.1) | 28,417 (54.2) | 35 (38.0) |
| Previous smoker | 18,305 (34.8) | 18,265 (34.8) | 40 (43.5) |
| Current smoker | 5,540 (10.5) | 5,523 (10.5) | 17 (18.5) |
| Missing | 255 (0.5) | 255 (0.5) | 0 (0) |
| Alcohol drinking (n, %) |  |  |  |
| Never drinking | 2,484 (4.7) | 2,479 (4.7) | 5 (5.4) |
| Previous drinking | 2,037 (3.9) | 2,029 (3.9) | 8 (8.7) |
| Current drinking | 47,894 (91.1) | 47,815 (91.1) | 79 (85.9) |
| Missing | 137 (0.3) | 137 (0.3) | 0 (0) |
| Physical exercise (n, %) |  |  |  |
| No | 15,176 (28.9) | 15,142 (28.9) | 34 (37.0) |
| Yes | 36,063 (68.6) | 36,010 (68.6) | 53 (57.6) |
| Missing | 1,313 (2.5) | 1,308 (2.5) | 5 (5.4) |
| Family history of cancer (n, %) |  |  |  |
| No | 34,188 (65.0) | 34,139 (65) | 49 (53.0) |
| Yes | 18,364 (35.0) | 18,321 (35) | 43 (47.0) |
| Cardiovascular disease at baseline (n, %) |  |  |  |
| No | 49,828 (94.8) | 49,746 (94.8) | 82 (89.0) |
| Yes | 2,724 (5.2) | 2,714 (5.2) | 10 (11.0) |
| Type 2 diabetes mellitus at baseline (n, %) |  |  |  |
| No | 51,417 (97.8) | 51,328 (97.8) | 89 (96.7) |
| Yes | 1,135 (2.2) | 1,132 (2.2) | 3 (3.3) |
| Hypertension at baseline (n, %) |  |  |  |
| No | 47,876 (91.1) | 47,799 (91.1) | 77 (84.0) |
| Yes | 4,676 (8.9) | 4,661 (8.9) | 15 (16.0) |
|  | | | |

Table S3 Single nucleotide polymorphisms associated with gastric cancer at a genome-wide significant level from the Finngen R12 dataset

| SNP | chrom | pos | ref | alt | nearest genes | *P*-value | beta | sebeta |
| --- | --- | --- | --- | --- | --- | --- | --- | --- |
| rs10216533 | 8 | 1.43E+08 | G | A | PSCA | 6.89E-12 | 0.202997 | 0.029592 |
| rs1045531 | 8 | 1.43E+08 | C | A | PSCA | 6.93E-12 | 0.202979 | 0.029593 |
| rs1045547 | 8 | 1.43E+08 | T | G | PSCA | 6.62E-12 | 0.203148 | 0.029589 |
| rs1045574 | 8 | 1.43E+08 | G | A | PSCA | 6.89E-12 | 0.202994 | 0.029592 |
| rs1045605 | 8 | 1.43E+08 | C | G | PSCA | 6.93E-12 | 0.202979 | 0.029593 |
| rs1048831 | 8 | 1.43E+08 | G | A | LY6K | 3.88E-13 | -0.2182 | 0.030056 |
| rs12134456 | 1 | 1.56E+08 | C | G | GON4L | 4.15E-08 | -0.16962 | 0.030927 |
| rs1307901506 | 8 | 1.43E+08 | CTT | C | LY6K | 7.92E-13 | 0.211607 | 0.029543 |
| rs13249440 | 8 | 1.43E+08 | G | A | JRK, PSCA | 1.81E-11 | 0.198931 | 0.0296 |
| rs13256647 | 8 | 1.43E+08 | C | T | JRK, PSCA | 1.81E-11 | 0.198931 | 0.0296 |
| rs13264644 | 8 | 1.43E+08 | A | G | JRK, PSCA | 9.26E-09 | -0.17724 | 0.030858 |
| rs13272904 | 8 | 1.43E+08 | T | C | LY6K | 1.40E-11 | -0.20385 | 0.030166 |
| rs1435453 | 8 | 1.43E+08 | C | T | LY6K | 3.97E-11 | -0.19836 | 0.030031 |
| rs1469811 | 8 | 1.43E+08 | G | A | LNCOC1 | 5.94E-13 | -0.21661 | 0.030077 |
| rs1529865 | 8 | 1.43E+08 | C | T | LNCOC1 | 1.02E-11 | -0.20552 | 0.030208 |
| rs1560986 | 8 | 1.43E+08 | T | C | LNCOC1 | 1.02E-11 | -0.20552 | 0.030208 |
| rs1594999 | 8 | 1.43E+08 | G | A | LNCOC1 | 5.86E-13 | -0.21667 | 0.030077 |
| rs1836633 | 8 | 1.43E+08 | G | A | LY6K | 3.31E-11 | -0.19913 | 0.030026 |
| rs2082801 | 8 | 1.43E+08 | A | G | LNCOC1, LY6K | 1.04E-11 | -0.20545 | 0.030208 |
| rs2164307 | 8 | 1.43E+08 | A | T | LNCOC1 | 1.04E-11 | -0.20545 | 0.030208 |
| rs2164308 | 8 | 1.43E+08 | C | T | LNCOC1, LY6K | 5.78E-13 | -0.21669 | 0.030072 |
| rs2244152 | 8 | 1.43E+08 | C | A | LNCOC1, LY6K | 5.74E-13 | -0.21671 | 0.030072 |
| rs2244163 | 8 | 1.43E+08 | T | G | LNCOC1, LY6K | 1.03E-11 | -0.20545 | 0.030204 |
| rs2257796 | 8 | 1.43E+08 | T | C | THEM6 | 3.29E-10 | -0.18791 | 0.0299 |
| rs2257840 | 8 | 1.43E+08 | C | T | THEM6 | 6.22E-10 | -0.18552 | 0.029996 |
| rs2294008 | 8 | 1.43E+08 | C | T | JRK, PSCA | 7.35E-12 | 0.202716 | 0.02959 |
| rs2294010 | 8 | 1.43E+08 | A | G | JRK, PSCA | 6.90E-12 | 0.202999 | 0.029593 |
| rs2376491 | 8 | 1.43E+08 | G | A | LNCOC1 | 1.05E-11 | -0.20539 | 0.030209 |
| rs2572898 | 8 | 1.43E+08 | G | C | LY6K | 1.35E-11 | -0.20401 | 0.030167 |
| rs2572899 | 8 | 1.43E+08 | A | G | LNCOC1, LY6K | 5.77E-13 | -0.21669 | 0.030072 |
| rs2572902 | 8 | 1.43E+08 | A | G | LNCOC1 | 5.85E-13 | -0.21667 | 0.030077 |
| rs2572903 | 8 | 1.43E+08 | A | G | LNCOC1 | 1.02E-11 | -0.20552 | 0.030208 |
| rs2572904 | 8 | 1.43E+08 | A | C | LNCOC1 | 5.86E-13 | -0.21666 | 0.030077 |
| rs2572905 | 8 | 1.43E+08 | C | T | LNCOC1 | 1.03E-11 | -0.20549 | 0.030206 |
| rs2572906 | 8 | 1.43E+08 | G | A | LNCOC1 | 1.20E-11 | -0.20477 | 0.030201 |
| rs2572907 | 8 | 1.43E+08 | G | A | LNCOC1 | 1.23E-11 | -0.20462 | 0.030196 |
| rs2572908 | 8 | 1.43E+08 | A | G | LNCOC1 | 1.20E-11 | -0.20474 | 0.030196 |
| rs2572909 | 8 | 1.43E+08 | C | G | LNCOC1 | 6.05E-13 | -0.21655 | 0.030079 |
| rs2572910 | 8 | 1.43E+08 | C | G | PSCA | 1.16E-11 | 0.200842 | 0.029602 |
| rs2585135 | 8 | 1.43E+08 | A | G | THEM6 | 8.98E-12 | 0.206189 | 0.030224 |
| rs2585136 | 8 | 1.43E+08 | A | G | THEM6 | 7.44E-12 | -0.2069 | 0.030209 |
| rs2585138 | 8 | 1.43E+08 | C | T | CTD-2292P10.2, THEM6 | 3.98E-10 | -0.18703 | 0.029902 |
| rs2585139 | 8 | 1.43E+08 | C | G | LNCOC1 | 1.09E-11 | -0.20525 | 0.030209 |
| rs2585140 | 8 | 1.43E+08 | A | G | LNCOC1 | 5.85E-13 | -0.21666 | 0.030075 |
| rs2585144 | 8 | 1.43E+08 | A | T | LNCOC1 | 6.92E-13 | -0.21591 | 0.030067 |
| rs2585145 | 8 | 1.43E+08 | G | A | LNCOC1 | 6.38E-13 | -0.21625 | 0.030068 |
| rs2585148 | 8 | 1.43E+08 | C | G | LNCOC1 | 1.38E-11 | -0.20422 | 0.030211 |
| rs2585149 | 8 | 1.43E+08 | T | C | LNCOC1 | 5.91E-13 | -0.21663 | 0.030077 |
| rs2585150 | 8 | 1.43E+08 | C | T | LNCOC1 | 5.87E-13 | -0.21666 | 0.030077 |
| rs2585151 | 8 | 1.43E+08 | G | T | LNCOC1 | 1.02E-11 | -0.20552 | 0.030208 |
| rs2585152 | 8 | 1.43E+08 | A | T | LNCOC1, LY6K | 5.95E-13 | -0.21661 | 0.030078 |
| rs2585153 | 8 | 1.43E+08 | G | A | LNCOC1, LY6K | 5.76E-13 | -0.2167 | 0.030072 |
| rs2585154 | 8 | 1.43E+08 | C | T | LNCOC1, LY6K | 1.07E-11 | -0.20533 | 0.03021 |
| rs2585174 | 8 | 1.43E+08 | A | G | LY6K | 5.92E-13 | -0.21631 | 0.030033 |
| rs2585175 | 8 | 1.43E+08 | G | C | LY6K | 1.95E-12 | -0.21078 | 0.029948 |
| rs2585176 | 8 | 1.43E+08 | A | T | LY6K | 1.05E-12 | 0.21049 | 0.029546 |
| rs2585179 | 8 | 1.43E+08 | G | A | LY6K | 1.16E-11 | 0.200868 | 0.029606 |
| rs2585181 | 8 | 1.43E+08 | C | A | PSCA | 1.05E-11 | 0.201357 | 0.029612 |
| rs2585183 | 8 | 1.43E+08 | C | G | PSCA | 6.63E-13 | 0.212446 | 0.029561 |
| rs2717550 | 8 | 1.43E+08 | T | A | LNCOC1 | 5.95E-13 | -0.21661 | 0.030078 |
| rs2717552 | 8 | 1.43E+08 | T | C | LNCOC1 | 5.85E-13 | -0.21667 | 0.030077 |
| rs2717600 | 8 | 1.43E+08 | A | G | THEM6 | 3.69E-11 | 0.201481 | 0.030453 |
| rs2717601 | 8 | 1.43E+08 | T | C | LNCOC1 | 3.31E-10 | -0.18786 | 0.029898 |
| rs2717602 | 8 | 1.43E+08 | G | A | LNCOC1 | 9.52E-12 | -0.20581 | 0.030205 |
| rs2717603 | 8 | 1.43E+08 | T | C | LNCOC1 | 6.79E-13 | -0.21601 | 0.03007 |
| rs2717605 | 8 | 1.43E+08 | T | G | LNCOC1 | 1.20E-11 | -0.20473 | 0.030196 |
| rs2717606 | 8 | 1.43E+08 | G | A | LNCOC1 | 6.94E-13 | -0.2159 | 0.030067 |
| rs2717608 | 8 | 1.43E+08 | A | C | PSCA | 1.33E-11 | 0.200372 | 0.029616 |
| rs2717609 | 8 | 1.43E+08 | A | T | PSCA | 1.00E-10 | 0.19304 | 0.029853 |
| rs2920279 | 8 | 1.43E+08 | A | C | JRK,PSCA | 6.90E-12 | 0.203 | 0.029593 |
| rs2920280 | 8 | 1.43E+08 | G | C | JRK,PSCA | 4.27E-13 | 0.214057 | 0.029539 |
| rs2920281 | 8 | 1.43E+08 | C | T | JRK,PSCA | 4.40E-13 | 0.213983 | 0.029545 |
| rs2920282 | 8 | 1.43E+08 | T | C | JRK,PSCA | 1.79E-11 | 0.198962 | 0.029599 |
| rs2920283 | 8 | 1.43E+08 | T | C | JRK,PSCA | 1.77E-11 | 0.199011 | 0.029599 |
| rs2920284 | 8 | 1.43E+08 | A | G | JRK,PSCA | 1.81E-11 | 0.198934 | 0.0296 |
| rs2920285 | 8 | 1.43E+08 | G | C | JRK,PSCA | 1.81E-11 | 0.198938 | 0.029601 |
| rs2920286 | 8 | 1.43E+08 | G | A | JRK,PSCA | 1.81E-11 | 0.198956 | 0.029604 |
| rs2920288 | 8 | 1.43E+08 | C | T | JRK,PSCA | 5.01E-11 | -0.19718 | 0.030009 |
| rs2920292 | 8 | 1.43E+08 | A | G | PSCA | 1.16E-11 | 0.200836 | 0.029601 |
| rs2920293 | 8 | 1.43E+08 | C | G | PSCA | 1.16E-11 | 0.200836 | 0.029601 |
| rs2920294 | 8 | 1.43E+08 | C | G | PSCA | 6.92E-12 | 0.202986 | 0.029593 |
| rs2920295 | 8 | 1.43E+08 | A | G | PSCA | 8.95E-12 | 0.201959 | 0.029602 |
| rs2920296 | 8 | 1.43E+08 | A | G | JRK,PSCA | 4.96E-12 | 0.204382 | 0.029592 |
| rs2920297 | 8 | 1.43E+08 | A | G | JRK,PSCA | 6.89E-12 | 0.203003 | 0.029593 |
| rs2920298 | 8 | 1.43E+08 | A | G | JRK,PSCA | 6.86E-12 | 0.203017 | 0.029592 |
| rs2976384 | 8 | 1.43E+08 | T | C | JRK,PSCA | 1.59E-11 | 0.199634 | 0.029622 |
| rs2976386 | 8 | 1.43E+08 | G | A | JRK,PSCA | 2.29E-11 | 0.197996 | 0.029612 |
| rs2976387 | 8 | 1.43E+08 | G | A | JRK,PSCA | 3.82E-13 | 0.214467 | 0.029533 |
| rs2976388 | 8 | 1.43E+08 | G | A | JRK,PSCA | 3.82E-13 | 0.214464 | 0.029533 |
| rs2976389 | 8 | 1.43E+08 | T | C | JRK,PSCA | 7.92E-12 | 0.202451 | 0.029598 |
| rs2976391 | 8 | 1.43E+08 | C | A | JRK,PSCA | 1.99E-11 | -0.2013 | 0.030013 |
| rs2976392 | 8 | 1.43E+08 | G | A | JRK,PSCA | 6.86E-12 | 0.203017 | 0.029592 |
| rs2976393 | 8 | 1.43E+08 | C | G | PSCA | 6.91E-12 | 0.202994 | 0.029593 |
| rs2976394 | 8 | 1.43E+08 | C | T | PSCA | 6.89E-12 | 0.203001 | 0.029593 |
| rs2976395 | 8 | 1.43E+08 | G | A | PSCA | 6.92E-12 | 0.202985 | 0.029593 |
| rs2976396 | 8 | 1.43E+08 | G | A | PSCA | 6.89E-12 | 0.202996 | 0.029592 |
| rs2976397 | 8 | 1.43E+08 | G | T | PSCA | 4.57E-13 | 0.213768 | 0.029536 |
| rs2976398 | 8 | 1.43E+08 | G | C | PSCA | 1.03E-11 | 0.201961 | 0.029689 |
| rs2978978 | 8 | 1.43E+08 | T | C | JRK,PSCA | 1.81E-11 | 0.198919 | 0.0296 |
| rs2978979 | 8 | 1.43E+08 | C | A | JRK,PSCA | 1.81E-11 | 0.19893 | 0.029599 |
| rs2978980 | 8 | 1.43E+08 | T | G | JRK,PSCA | 1.81E-11 | 0.198928 | 0.029599 |
| rs2978981 | 8 | 1.43E+08 | C | T | JRK,PSCA | 5.21E-12 | 0.204226 | 0.029599 |
| rs2978982 | 8 | 1.43E+08 | T | C | PSCA | 6.90E-12 | 0.202999 | 0.029593 |
| rs2978983 | 8 | 1.43E+08 | A | G | PSCA | 2.27E-11 | -0.2009 | 0.030042 |
| rs2990223 | 1 | 1.55E+08 | G | A | GBAP1 | 1.25E-08 | -0.17724 | 0.031138 |
| rs34635647 | 8 | 1.43E+08 | A | G | JRK,PSCA | 6.19E-11 | 0.193637 | 0.029613 |
| rs372173246 | 8 | 1.43E+08 | G | C | JRK,PSCA | 1.63E-13 | 0.220502 | 0.029895 |
| rs4971091 | 1 | 1.55E+08 | G | T | KRTCAP2,RP11-201K10.3 | 3.51E-08 | 0.163906 | 0.029726 |
| rs4971093 | 1 | 1.55E+08 | G | A | KRTCAP2,RP11-201K10.3 | 3.56E-08 | 0.163841 | 0.029727 |
| rs6676150 | 1 | 1.55E+08 | G | C | HMGN2P18 | 1.65E-08 | -0.17581 | 0.031143 |
| rs760077 | 1 | 1.55E+08 | T | A | MTX1,THBS3 | 1.61E-08 | -0.17588 | 0.03113 |
| rs9297976 | 8 | 1.43E+08 | T | C | JRK,PSCA | 7.09E-09 | -0.17645 | 0.030481 |

Table S4 Proteins associated with gastric cancer in both discovery and validation stages

| Protein | Discovery set | | |  | Validation set | |  |
| --- | --- | --- | --- | --- | --- | --- | --- |
|  | Log_2_FC | *P* value | FDR |  | HR (95%CI) | *P* value | FDR |
| CTSD | 0.93 | 3.94×10^-16^ | 2.58×10^-14^ |  | 2.02(1.50-2.74) | 5.01×10^-6^ | 5.45×10^-4^ |
| KRT19 | 1.09 | 2.31×10^-12^ | 7.44×10^-11^ |  | 1.46(1.14-1.85) | 2.27×10^-3^ | 1.06×10^-1^ |
| GGH | 0.75 | 2.62×10^-12^ | 8.34×10^-11^ |  | 2.60(1.84-3.67) | 6.56×10^-8^ | 2.14×10^-5^ |
| PIGR | 0.93 | 7.64×10^-10^ | 1.58×10^-8^ |  | 1.88(1.21-2.92) | 4.90×10^-3^ | 1.23×10^-1^ |
| ITGA11 | -2.36 | 3.64×10^-8^ | 5.37×10^-7^ |  | 0.53(0.32-0.90) | 1.91×10^-2^ | 2.08×10^-1^ |
| FABP5 | 0.38 | 4.55×10^-7^ | 5.56×10^-6^ |  | 1.46(1.14-1.87) | 2.97×10^-3^ | 1.12×10^-1^ |
| CTRC | -0.74 | 9.66×10^-7^ | 1.12×10^-5^ |  | 0.68(0.52-0.90) | 5.76×10^-3^ | 1.26×10^-1^ |
| MMP7 | 1.86 | 1.72×10^-6^ | 1.93×10^-5^ |  | 1.65(1.12-2.42) | 1.17×10^-2^ | 1.74×10^-1^ |
| SERPINA12 | 0.53 | 6.73×10^-6^ | 6.26×10^-5^ |  | 1.17(1.02-1.33) | 2.36×10^-2^ | 2.26×10^-1^ |
| PTN | 0.74 | 4.27×10^-5^ | 2.65×10^-4^ |  | 1.44(1.02-2.05) | 3.95×10^-2^ | 2.69×10^-1^ |
| GGT5 | -0.75 | 8.09×10^-5^ | 4.45×10^-4^ |  | 0.42(0.19-0.94) | 3.37×10^-2^ | 2.51×10^-1^ |
| TNFRSF14 | 0.27 | 1.90×10^-4^ | 8.93×10^-4^ |  | 1.81(1.06-3.10) | 3.06×10^-2^ | 2.51×10^-1^ |
| PSIP1 | 1.01 | 2.73×10^-4^ | 1.21×10^-3^ |  | 1.29(1.04-1.60) | 2.18×10^-2^ | 2.22×10^-1^ |
| NCF2 | 1.07 | 3.74×10^-4^ | 1.54×10^-3^ |  | 1.25(1.05-1.50) | 1.45×10^-2^ | 1.89×10^-1^ |
| SCGB3A2 | 1.11 | 7.22×10^-4^ | 2.70×10^-3^ |  | 1.22(1.02-1.46) | 3.39×10^-2^ | 2.51×10^-1^ |
| GYS1 | 1.26 | 8.68×10^-4^ | 3.15×10^-3^ |  | 1.24(1.02-1.51) | 3.02×10^-2^ | 2.51×10^-1^ |
| PON3 | -0.35 | 8.92×10^-4^ | 3.21×10^-3^ |  | 0.64(0.44-0.92) | 1.59×10^-2^ | 1.91×10^-1^ |
| PVALB | 4.54 | 1.25×10^-3^ | 4.26×10^-3^ |  | 1.20(1.01-1.44) | 3.79×10^-2^ | 2.69×10^-1^ |
| PARP1 | 1.54 | 1.87×10^-3^ | 5.96×10^-3^ |  | 1.30(1.05-1.60) | 1.78×10^-2^ | 2.07×10^-1^ |
| SFRP1 | 3.58 | 1.87×10^-3^ | 5.96×10^-3^ |  | 1.45(1.00-2.11) | 4.71×10^-2^ | 3.01×10^-1^ |
| ATP6AP2 | -0.28 | 1.04×10^-2^ | 2.41×10^-2^ |  | 0.50(0.28-0.89) | 1.89×10^-2^ | 2.08×10^-1^ |
| F2R | 0.19 | 1.06×10^-2^ | 2.46×10^-2^ |  | 1.43(1.04-1.96) | 2.87×10^-2^ | 2.51×10^-1^ |
| LSP1 | 0.47 | 1.13×10^-2^ | 2.59×10^-2^ |  | 1.77(1.18-2.66) | 5.80×10^-3^ | 1.26×10^-1^ |
| LGALS1 | 0.47 | 1.86×10^-2^ | 3.94×10^-2^ |  | 1.62(1.01-2.61) | 4.53×10^-2^ | 2.96×10^-1^ |
| ALDH3A1 | 1.62 | 2.38×10^-2^ | 4.84×10^-2^ |  | 1.36(1.11-1.66) | 3.09×10^-3^ | 1.12×10^-1^ |

FC, fold change; FDR, false discovery rate; HR, hazard ratio.

Table S5 The hazard ration and 95% confidence interval for variables included in the clinical risk prediction model in training set

| Predictors | HR | 95%CI | *P* |
| --- | --- | --- | --- |
| Age | 1.08 | 1.04-1.11 | **＜0.001** |
| Sex |  |  |  |
| Female | Ref |  |  |
| Male | 2.87 | 1.81-4.54 | **＜0.001** |
| Education |  |  |  |
| Below College | Ref |  |  |
| College or University degree | 0.54 | 0.31-0.95 | **0.031** |
| Unknown | 0.56 | 0.35-0.90 | **0.018** |
| Smoking status |  |  |  |
| Never | Ref |  |  |
| Previous | 1.32 | 0.83-2.09 | 0.237 |
| Current | 2.30 | 1.27-4.14 | **0.006** |
| Unknown | 0.00 | 0.00-Inf | 0.993 |
| Physical activity |  |  |  |
| No | Ref |  |  |
| Yes | 0.67 | 0.44-1.04 | 0.075 |
| Unknown | 1.45 | 0.56-3.74 | 0.447 |
| Family history of cancer |  |  |  |
| No | Ref |  |  |
| Yes | 1.63 | 1.07-2.46 | **0.022** |

HR, hazard ratio; CI, confidence interval.
